# Supplementary material for: End Sequence Analysis Toolkit (ESAT) expands the extractable information from single-cell RNA-seq data
Source: Genome Res. 2016 Oct;26(10):1397–410. doi: 10.1101/gr.207902.116 (PMC5052061; doi:10.1101/gr.207902.116)
Supplement: Supplemental Material [file supp_gr.207902.116_Supp_Materials.tar.gz › ESAT-esat_v0.1/doc/html/classbroad_1_1pda_1_1gene_1_1_neighbors_null_model-members.html]

ESAT: Member List


ESAT


- Main Page
- Related Pages
- Packages
- Classes
- Files

- Class List
- Class Index
- Class Hierarchy
- Class Members

All Classes Namespaces Files Functions Variables Enumerator Pages

- broad
- pda
- gene
- NeighborsNullModel

broad.pda.gene.NeighborsNullModel Member List

This is the complete list of members for broad.pda.gene.NeighborsNullModel, including all inherited members.

|  |  |  |
| --- | --- | --- |
| getGeneRandNeighbors(Gene gene) | broad.pda.gene.NeighborsNullModel |  |
| getGeneRandNeighbors() | broad.pda.gene.NeighborsNullModel |  |
| getPermNum() | broad.pda.gene.NeighborsNullModel |  |
| getUniqueNeighbors() | broad.pda.gene.NeighborsNullModel |  |
| makeBinnedIntervalModel(boolean UseCustomBins) | broad.pda.gene.NeighborsNullModel |  |
| makeDartThrowingModel() | broad.pda.gene.NeighborsNullModel |  |
| makeNaiveRandModel(String chrSizeF) | broad.pda.gene.NeighborsNullModel |  |
| NeighborsNullModel() | broad.pda.gene.NeighborsNullModel |  |
| NeighborsNullModel(String setname, String refname, int binsize, int permnum) | broad.pda.gene.NeighborsNullModel |  |
| NeighborsNullModel(BEDFileParser setname, BEDFileParser refname, int binsize, int permnum) | broad.pda.gene.NeighborsNullModel |  |
| printRandNeighborsInfo() | broad.pda.gene.NeighborsNullModel |  |


---

Generated on Fri Aug 15 2014 15:11:00 for ESAT by  

 1.8.7
